# Predictive Factors for Pathologic Complete Response Following Neoadjuvant Chemoradiotherapy for Rectal Cancer

**DOI:** 10.31557/APJCP.2021.22.5.1607

**Published:** 2021-05

**Authors:** Qi Zhang, Jianwei Liang, Jianan Chen, Shiwen Mei, Zheng Wang

**Affiliations:** *Department of Colorectal Surgery, National Cancer Center/National Clinical Research Center for Cancer/Cancer Hospital, Chinese Academy of Medical Sciences and Peking Union Medical College, China. *

**Keywords:** Rectal cancer, pathologic complete response, neoadjuvant chemoradiotherapy, CEA, interval

## Abstract

**Background:**

An accurate assessment of potential pathologic complete response(pCR) following neoadjuvant chemoradiotherapy(NCRT) is important for the appropriate treatment of rectal cancer. However, the factors that predict the response to neoadjuvant chemoradiotherapy have not been well defined. Therefore, this study analyzed the predictive factors on the development of pCR after neoadjuvant chemoradiation for rectal cancer.

**Methods:**

From January 2008 to January 2018, a total of 432 consecutive patients from a single institution patients who underwent a long-course neoadjuvant chemoradiotherapy were reviewed in this study. The clinicopathological features were analyzed to identify predictive factors for pathologic complete response in rectal cancer after neoadjuvant chemoradiation.

**Results:**

The rate of pathologic complete response in rectal cancer after neoadjuvant chemoradiation was 20.8%, patients were divided into the pCR and non-pCR groups. The two groups were well balanced in terms of age, gender, body mass index, ASA score, tumor stage, tumor differentiation, tumor location, surgical procedure, chemotherapy regimen and radiation dose. The multivariate analysis revealed that a pretreatment carcinoembryonic antigen (CEA) level of ≤5 ng/mL and an interval of ≥8 weeks between the completion of chemoradiation and surgical resection were independent risk factors of an increased rate of pCR.

**Conclusions:**

Pretreatment carcinoembryonic antigen (CEA) level of ≤5 ng/mL and an interval of ≥8 weeks between the completion of chemoradiation and surgical resection are predictive factors for pathologic complete response in rectal cancer after neoadjuvant chemoradiation. Using these predictive factors, we can predict the prognosis of patients and develop adaptive treatment strategies. A wait-and-see policy might be possible in highly selective cases.

## Introduction

Neoadjuvant chemoradiotherapy (NCRT) and total mesorectal excision (TME) have been shown to significantly decrease the local recurrence rate and improve the overall survival (OS) rate (Sauer et al., 2004; Sauer et al., 2012), which have become the standard of care for patients with clinical stages II and III rectal cancer (Fleming et al., 2011). Neoadjuvant chemoradiotherapy also can increases the rate of sphincter preservation (Glimelius, 2013). Most patients respond to neoadjuvant chemoradiotherapy. After neoadjuvant chemoradiotherapy, about 10%-30% of the patients get complete pathological response, and there is no viable tumor cells in the surgical specimens (Martin et al., 2012). However, some patients did not respond to neoadjuvant chemoradiotherapy. Recent studies have shown that the prognosis of rectal cancer patients with pathological complete response after neoadjuvant chemoradiotherapy is better than that without pathological complete response (Maas et al., 2010a; de Campos-Lobato et al., 2011b). A wait-and-see policy might be a consideration in rectal cancer patients treated with neoadjuvant chemoradiotherapy and who have already clinical complete responded to treatment (Habr-Gama et al., 2004; Maas et al., 2011).

Patients with ypT0N0 rectal cancer are a subgroup that responds well to neoadjuvant chemoradiotherapy and have favorable oncological prognosis, with a 5-year disease-free survival (DFS) rate reaching 83%-95% (Stipa et al., 2006; Capirci et al., 2008; Maas et al., 2010b; Zorcolo et al., 2012; Wasmuth et al., 2016). So, the ability to predict the efficacy of neoadjuvant chemoradiotherapy for rectal cancer has important clinical significance. However, it is very difficult to predict the pathological complete response of rectal cancer after neoadjuvant chemoradiotherapy, and the relevant research is limited. 

The present retrospective study was designed to evaluate the clinical factors that can be predicted pathologic complete response to neoadjuvant chemoradiotherapy for rectal cancer.

## Materials and Methods

*Patients and methods*

*Patients and evaluation before the treatment*

This retrospective study was conducted at the Department of Colorectal Surgery, National Cancer Center/National Clinical Research Center for Cancer/Cancer Hospital, Chinese Academy of Medical Sciences and Peking Union Medical College, Beijing, China. The study was performed in accordance with the ethical standards of our institutional research committee, and the principles of the 1964 Declaration of Helsinki and its later amendments. Informed consent was obtained from each individual participant included in the study. The data of 432 consecutive patients with rectal cancer who received neoadjuvant chemoradiotherapy in our hospital from January 2008 to January 2018 were retrospectively analyzed. The criteria for neoadjuvant chemoradiotherapy were as follows: (I) low or middle rectal carcinoma (a distance of <10 cm between the inferior tumor edge and the anal verge); (II) pretreatment clinical stage was II and III; (III) no obvious distant metastasis; (IV) postoperative pathological results showed R0 resection. The exclusion criteria were: (I) other malignant tumors present (except for rectal cancers); (II) a history of malignant tumor or relapse; (III) managed by a watch-and-wait strategy after neoadjuvant chemoradiotherapy.

All patients underwent colonoscopy with biopsy and were histologically diagnosed with adenocarcinoma. Preoperative clinical staging was determined by abdominal and pelvic computed tomography (CT), transrectal ultrasonography, pelvic magnetic resonance imaging, or a combination of these. Postoperative specimens were examined by at least two pathologists specialized in colorectal cancer. The specimens were examined grossly and microscopically. pCR was defined as the absence of viable tumor cells in the surgical specimen, including lymph nodes. Patients without pCR were grouped into the non-pCR cohort. Clinicopathological classification and staging were based on the American Joint Committee on Cancer tumor-node-metastasis (TNM) staging system.

*Treatment*

All of the patients received intensity-modulated radiation therapy with concurrent oral administration of capecitabine: the total dosage was 50Gy (2.0 Gy per time, 25fractions). One of the two chemotherapeutic regimens was delivered concurrently with radiotherapy as follows: oral capecitabine at a dose of 1,650 mg/m^2^ per day for 35 days, without weekend breaks, or oral capecitabine at a dose of 1,650 mg/m^2^ per day for 35 days plus intravenous oxaliplatin at a dose of 50 mg/m^2^ once weekly for 5 weeks. Surgical resection was planned for 6-8weeks after the completion of neoadjuvant chemoradiotherapy, irrespective of the response to chemoradiotherapy. All patients underwent radical total mesorectal excision (TME).

The clinicopathological and operative data were documented, including age, gender, body mass index, ASA score, tumor stage, tumor differentiation, tumor location, surgical procedure, pretreatment serum CEA level, chemotherapy regimen, the interval between neoadjuvant chemoradiotherapy and surgery, and radiation dose. Data of last follow-up and vital status were collected on all patients. Each patient was followed-up every three months for the first two years, every six months for the next three years, and once a year thereafter. Digital rectal examination was performed and the levels of carcinoembryonic antigen (CEA) and carbohydrate antigen (CA) 19-9 were determined at every follow-up visit. Chest computed tomography, magnetic resonance imaging, or computed tomography with intravenous contrast of the liver and pelvis, and full colonoscopy were regularly undertaken. 

*Statistical analysis*

Nonparametric variables are presented as the median and range, and categorical variables are presented as the frequency with percentages. Continuous variables were analyzed with the Mann–Whitney U test, and categorical variables were analyzed with the Chisquare test or Fisher’s exact test, when appropriate. univariate and multivariate analyses were performed by using logistic regression and Cox proportional hazard ratios to identify factors that predict pCR. P<0.05 was considered statistically significant. Data were analyzed by Statistical Package for the Social Science (SPSS) for Windows (SPSS Inc., Chicago, IL, USA). 

## Results

Patient demographics and tumor characteristics are shown in Table 1. In our hospital, a total of 432 consecutive patients with clinical stages II and III rectal adenocarcinoma who underwent long-course neoadjuvant chemoradiotherapy followed by TME were included in this study. Of the 432 patients, 90 (20.8%) achieved a pCR, and 342 (79.2%) did not. The patients were divided into two groups, the pCR (n=90) and non-pCR (n=342) groups. The age, gender, body mass index, ASA score, tumor stage, tumor differentiation, tumor location, surgical procedure, chemotherapy regimen and radiation dose were not statistically different between the two groups.The pretreatment serum CEA level was significantly lower in the pCR group than in the non-pCR group (2.6 vs. 5.8 ng/mL, P=0.001). The median interval between the completion of neoadjuvant chemoradiotherapy and surgery was significantly longer in the pCR group than in the non pCR group (56 vs. 49 days, P< 0.001).

For the univariate and multivariate analyses, the variables were analyzed as discrete categorical variables. On multivariate analysis, a pretreatment CEA level of ≤5 ng/mL (OR=1.9, 95% CI=1.22-3.84, P=0.018) and an interval from the completion of neoadjuvant chemoradiotherapy to surgery of ≥8 weeks (OR=2.1, 95% CI=1.81-5.06, P=0.009) were identified as independent predictors for achieving a pCR (Table 2).

**Table 1:** Patient Demographics and Tumor Characteristics

Variable	pCR (n =90)	Non-pCR (n =342)	P value
Age (years)	60 (26–86)	59 (32–82)	0.812
Gender (male / female)	53/37	189/153	0.564
BMI(kg/m^2^)	24.6 (17.4–33.2)	24.5 (17.0–32.8)	0.462
ASA score			0.735
1	11 (12.2%)	51 (14.9%)	
2	46 (51.1%)	158 (46.2%)	
3	33 (36.7%)	133 (38.9%)	
cT category			0.258
2	9 (10.0)	30 (8.8)	
3	73 (81.1)	256 (74.9)	
4	8 (8.9)	56 (16.4)	
cN category			0.784
N0	26 (28.9)	96 (28.0)	
N1	44 (48.9)	169 (49.4)	
N2	20 (22.2)	77 (22.5)	
Tumor stage			0.884
II	28 (31.1)	96 (28.0)	
III	62 (68.9)	181 (71.9)	
Differentiation			0.715
Well	8 (8.9%)	28 (8.2%)	
Moderately	71 (78.9%)	265 (77.5%)	
Poor	11 (12.2%)	49 (14.3%)	
Distance of tumor from the anal verge (cm)	6 (0-10)	6 (0-10)	0.845
Pretreatment CEA (ng/mL)	2.6 (0.05-24.9)	5.8 (0.02–35.8)	0.001
Surgical procedure			0.539
Anterior resection	52 (57.8%)	208 (60.8%)	
Abdominoperineal resection	32 (35.6%)	120 (35.1%)	
Hartmann procedure	6 (6.7%)	14 (4.1%)	
Chemotherapy			0.712
Capecitabine	75 (83.3)	285 (83.3)	
Capecitabine + oxaliplatin	15 (16.7)	57 (16.7)	
Time interval (day)	56 (35-95)	49 (25-105)	<0.001
Radiation dose (Gy)	50 (40-60)	50 (38-60)	0.354

**Table 2:** Predictors for Pathologic Complete Response by Multivariate Analysis

Factor	OR	95% CI	P-value
Serum CEA (ng/mL) ≤5	1.9	1.22-3.84	0.018
Time interval (weeks)≥8	2.1	1.81-5.06	0.009

## Discussion

For clinical stages II and III rectal cancer, the standard treatment is neoadjuvant chemoradiotherapy and surgery. Compared with surgical resection alone or postoperative chemoradiotherapy, neoadjuvant chemoradiotherapy improves local control. The prognosis of patients with tumor regression and T or N downstaging after neoadjuvant chemoradiotherapy for rectal cancer has been shown to improve significantly. It has been well documented that patients who achieved pCR had better long-term outcomes than those without pCR. However, the factors that predict patient response to neoadjuvant chemoradiotherapy for rectal cancer have not been well defined. Some studies have evaluated clinical factors associated with complete response to preoperative chemoradiotherapy for rectal cancer (García-Aguilar et al., 2003; Das et al., 2007b; Kalady et al., 2009b; Park et al., 2011; Garland et al., 2014a). Armstrong et al., (2015) found that lower pretreatment CEA level, proximity to anal verge, and statin use were predictors of pCR after evaluating the clinical factors of 885 patients. Das et al., (2007a) identified that the circumferential tumor extent was the only factor that was significantly associated with pCR in a large sample size retrospective review of 562 patients. Kalady et al., (2009a) evaluated the predictors of pCR in 242 patients and found that an interval of >8 weeks between the completion of preoperative chemoradiotherapy and surgical resection was the only factor that was significantly associated with pCR. Garland et al., (2014b) found that the tumor size and pretreatment clinical N category were independent predictors of pCR after evaluating the clinical factors of 297 patients. In this analysis of a consecutive series of rectal cancer patients who underwent neoadjuvant chemoradiotherapy and radical resection from a single institution, the rate of postoperative pathologic complete response(pCR) was 20.8%, and we found that a pretreatment CEA level of ≤5 ng/mL and an interval from the completion of neoadjuvant chemoradiotherapy to surgery of ≥8 weeks were independent predictors of pCR. 

Carcinoembryonic antigen (CEA) is a tumor associated antigen, CEA has important clinical value in the condition monitoring and curative effect evaluation of colorectal cancer (Berman et al., 2000). In colon cancer, CEA levels have been shown to be an important prognostic factor in node-negative disease, with an increase of the preoperative serum CEA level associated with an increased risk of recurrence (Harrison et al., 1997). Consistent with our findings, Yeo et al., (2013) also showed that CEA level before treatment was an important predictive factor of pCR in a cohort of 609 patients who received preoperative chemoradiotherapy. Riesterer et al., (2007) found that tumor cells which have a high density of CEA may resist radiation. Garland et al., (2014c) found the pretreatment serum CEA levels and a decrease in the pre- to post-treatment serum CEA level were independent risk factors of pCR. Another recent study confirmed the value of CEA level before treatment on pCR, but found that the predictive value was limited to patients who were nonsmokers (Wallin et al., 2013). We found that the CEA level was significantly higher in the non-pCR group than in the pCR group, and a normal pretreatment CEA level was significantly associated with pCR in both univariate and multivariate analyses.

In 1999, François et al., (1999) (The Lyon Trial) advocated the adoption of an interval between chemoradiation and surgery of 6 to 8 weeks. This was based on a statistically nonsignificant improvement in sphincter preservation rates without changes in perioperative complications, compared to an interval of 2-3 weeks. Based on these equivocal findings, an interval between chemoradiation and surgery of 6 to 8 weeks has become part of the standard protocol for the treatment of rectal cancer in the USA. Radiation-induced necrosis and subsequent tumor regression is a time-dependent phenomenon in which a longer interval between the completion of chemoradiotherapy and surgery may increase the rate of pCR (Kalady et al., 2009c). Therefore, the persistent effects of neoadjuvant treatment would continue to cause cell death over time, and consequently, waiting longer before surgery could yield less viable carcinoma at the time of surgery. In this study, we found that a time interval between chemoradiation and surgery ≥8 weeks was the independent predictor of pCR(OR=2.1, 95% CI=1.81-5.06, P=0.020). Similarly, Kalady et al. reported that an interval of >8 weeks was an independent predictor for pCR (OR=2.63, 95% CI =1.13-6.12, P =0.020) (Kalady et al., 2009c). Wolthuis et al., (2012) reported that an interval of >7 weeks was associated with increased pCR (28 vs. 16%, P = 0.030), and the 5-year cancer-specific survival rate was higher in the long-interval group than in the short-interval group (91% vs. 83%, P=0.046). 

de Campos-Lobato et al., (2011a) has demonstrated that a prolonged interval between chemoradiation and surgery was an independent predictor of achieving a pCR, and this study shows that a longer interval is safe for patients as it does not increase peri or postoperative morbidity. Furthermore, an interval of greater than or equal to 8 weeks resulted in a decreased rate of local recurrence.

Despite attempts to improve tumor response by varying chemotherapy regimens, no significant impact on oncologic outcomes has been made. To improve the response, some randomized trials have added oxaliplatin or targeted drugs into the currently widely used fluorinated, pyrimidine-based preoperative chemotherapy regimen (Gérard et al., 2012; O’Connell et al., 2014). However, none of these studies reported an increased pCR rate. Our study found that the addition of oxaliplatin did not increase the pCR rate.

Due to the retrospective nature of this study, it is subject to potential bias and certain limitations. Firstly, this is a single-center, retrospective study. Besides, there were fewer patients in the pCR group than in the non-pCR group, there may be bias. Secondly, Since the study was not prospectively designed, the time interval between chemoradiation and surgery was decided according to the individual surgeon preference. To solve these controversial factors, randomized clinical trials should be performed in the future and identify the predictors for pCR and elucidate its potential mechanisms. Patients who are considered good responders to neoadjuvant chemoradiotherapy may benefit little from TME, and their optimal treatment may involve nonoperative management or local excision.

In conclusion, this large, retrospective study demonstrated that a pretreatment CEA level of ≤5 ng/mL and an interval from the completion of neoadjuvant chemoradiotherapy to surgery of ≥8 weeks were independent clinical predictors for achieving pCR. These findings may help clinicians predict the prognosis of patients and develop individualized treatment strategies.

## Author Contribution Statement

Zheng Wang and Qi Zhang contributed the conceiving and designing the study. Jianwei Liang and Jianan Chen did the collecting of data. Shiwen Mei did the analyzing and interpreting of data. Qi Zhang and Zheng Wang did the writing of the article. Zheng Wang approved the final version of the article. 
